# Socioeconomic and Regional Disparities in Industry-Sponsored Clinical Trials in Multiple Sclerosis

**DOI:** 10.1001/jamanetworkopen.2023.45619

**Published:** 2023-11-29

**Authors:** Stefanie Marti, Andreas G. F. Hoepner, Helly Hammer, Anke Salmen, Andrew Chan, Robert Hoepner

**Affiliations:** 1 Department of Neurology, Inselspital, Bern University Hospital, University of Bern, Bern, Switzerland; 2 Department of Banking and Finance, Michael Smurfit Graduate Business School, University College Dublin, Dublin, Ireland; 3 Department of Neurology, St Josef-Hospital Bochum, Ruhr-University Bochum, Bochum, Germany

## Abstract

This cross-sectional study examines the geographical and socioeconomic factors associated with trial distribution and outcome of treatment for multiple sclerosis (MS).

## Introduction

Addressing inequities in international industry-sponsored clinical trials of multiple sclerosis (MS) represents an unmet need. Doing so elucidates limitations in generalizability and moves toward a more inclusive process with equitable access to trials and treatments.^1^

## Methods

Trial information from ClinicalTrials.gov^2^ was combined with geographical and socioeconomic data from Natural Earth^3^ and human development data from the United Nations Statistics Division.^4^ Included were all phase 1 to 4 trials in MS, with the first trial in 1994 and registration through May 17, 2023, and with funder type industry, intervention type drug, study type interventional, and detailed location listing. Each location was counted as a trial site. Trials listing multiple phases were included for each phase separately. In accordance with the Common Rule, this cross-sectional study was exempt from review and informed consent because only deidentified publicly available data were used. We followed the STROBE reporting guideline.

Data processing and analyses were performed in Python 3.10 (Python Software) (eMethods in Supplement 1). The code is available on GitHub.

## Results

A total of 435 phase 1 to 4 trials sponsored by 94 companies were conducted in 78 of 195 countries worldwide. Most frequent sponsors were Biogen (80 trials [18.4%]), Novartis (47 [10.8%]), Sanofi (31 [7.1%]), Hoffman-La Roche (26 [6.0%]), and GlaxoSmithKline (16 [3.7%]), financing 200 trials (46.0%). At least 1 site per continent was registered in Africa (29 trials [6.7%]); Asia (140 [32.2%]); Europe (297 [68.3%]); North America (267 [61.4%]); Australia, including Oceania region (76 [17.5%]); and South America (54 [12.4%]).

**Figure 1.**
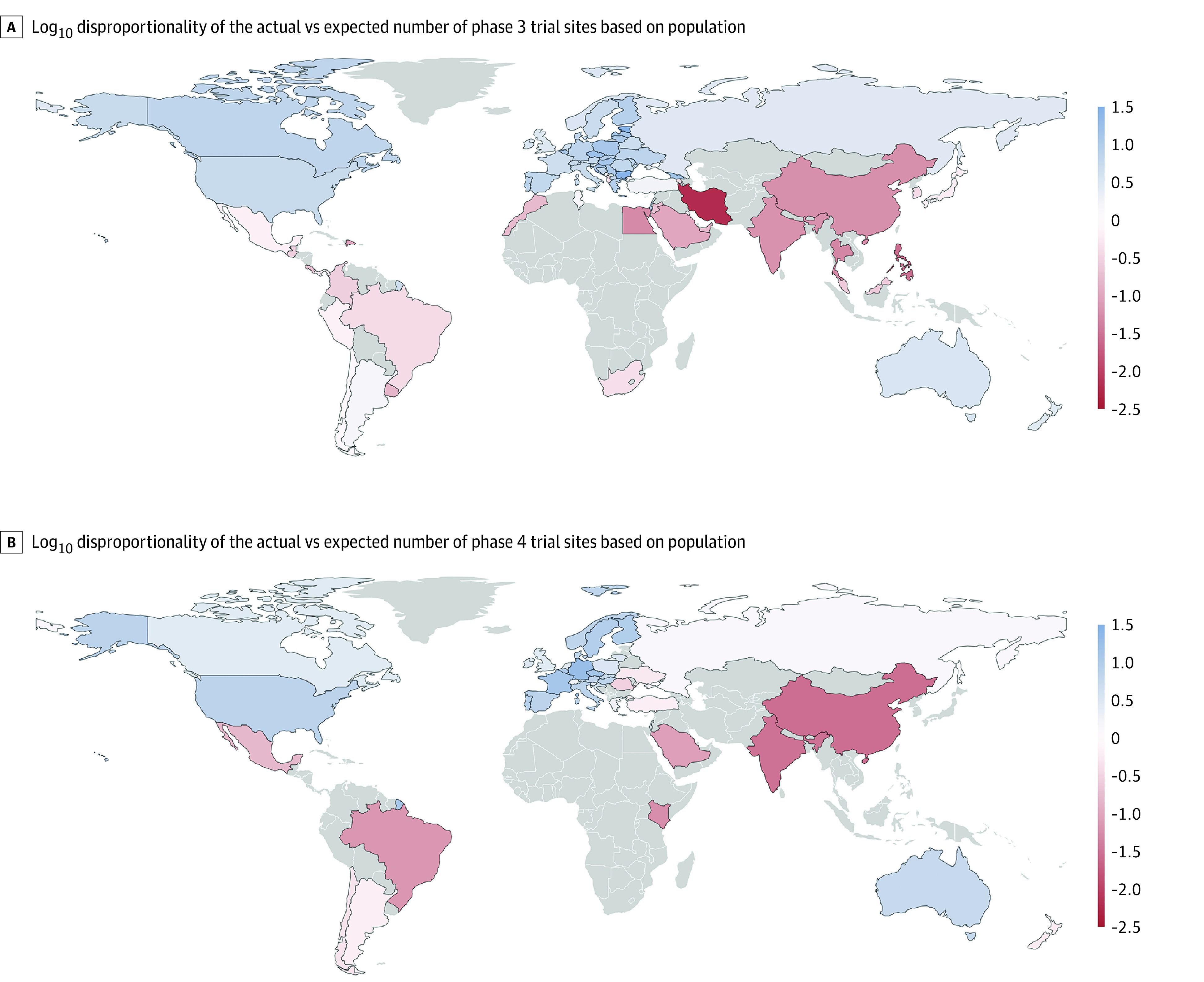
Disproportionality of Trial Sites Data on industry-funded interventional drug trials for multiple sclerosis were from ClinicalTrials.gov. For each country, the expected number of trial sites was computed under the assumption that the sites were equally distributed among the world population. Blue indicates that the actual number of sites was greater than expected, red indicates that the actual number was smaller than expected, and gray indicates no trial sites at all.

**Figure 2.**
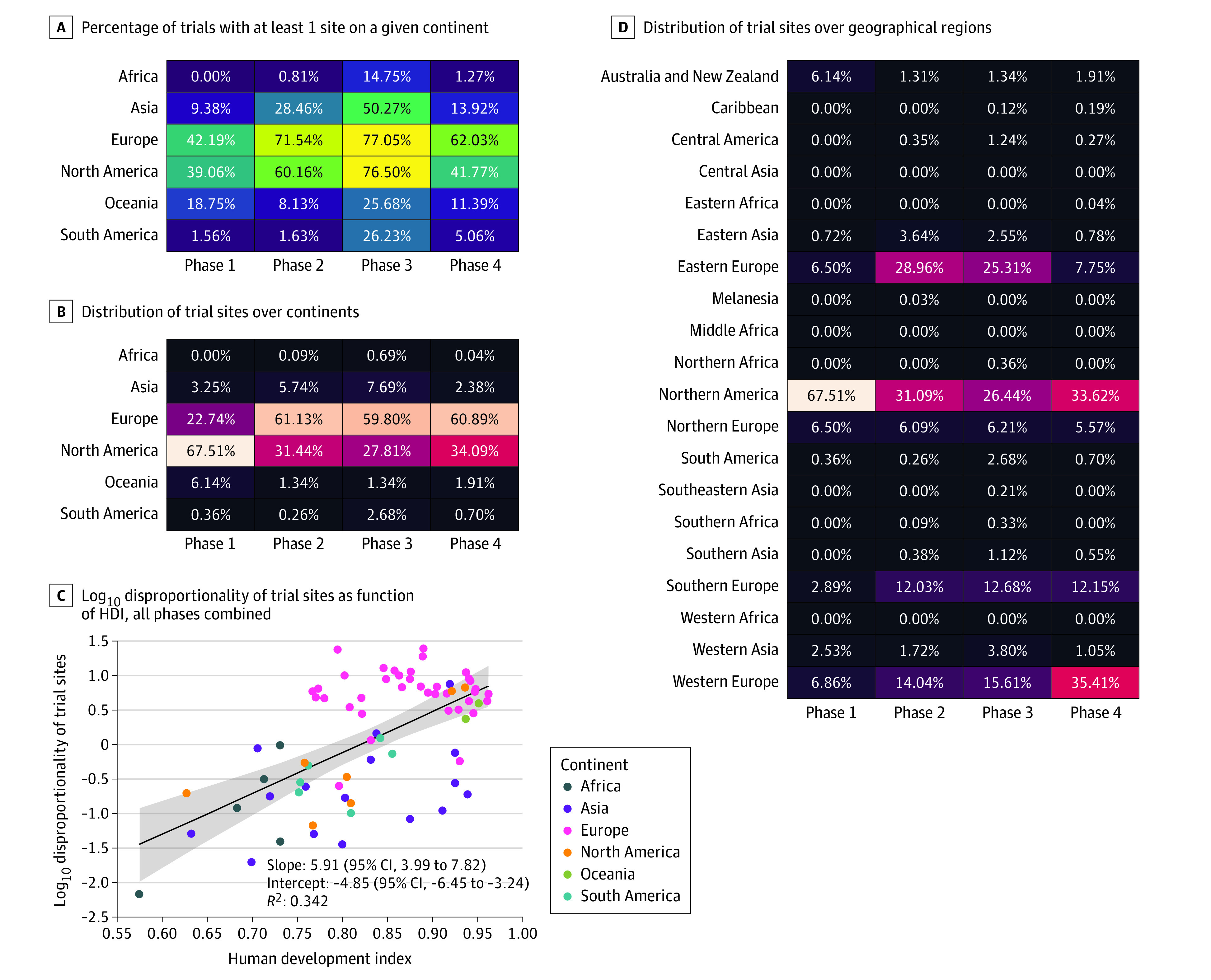
Distribution of Trial Sites A-D, Data on industry-funded interventional drug trials for multiple sclerosis were from ClinicalTrials.gov, and Human Development Index (HDI) data were from the United Nations. In panel A, color changes from dark blue to light yellow represent the the low to high percentages of trials. In panels B and D, color changes from black to light red represent the low to high percentages of trial sites. Details on linear regression for panel C are provided in the eMethods in Supplement 1.

Trial sites per capita ranged from 1.2 × 10^−8^ in Iran to 6.7 × 10^−5^ in Estonia (all phases combined). Phase 2 to 3 trial sites per population were highest in the Czech Republic (phase 2) and Estonia (phase 3), whereas the largest number of phase 4 trial sites per capita was found in Germany; the world maps of the log_10_ disproportionality of trial sites are provided in Figure 1. This log_10_ disproportionality seemed proportional to the Human Development Index (HDI) (Figure 2).

We observed a shift from Eastern to Western Europe from phases 2 and 3 (2.1 and 1.6 times more sites, respectively, in Eastern Europe) to phase 4 (4.6 times more sites in Western Europe) (Figure 2). Most trials were conducted in countries with very high HDI (89.0% of all sites) or high HDI (10.0%), while overlooking countries with medium HDI (1.0%) and low HDI (0%).

## Discussion

Multiple sclerosis, a chronic neurological disease with comparatively high medication costs, was chosen as the model disease. The findings showed inequality in trial distribution over geographical regions and socioeconomic strata, with phase 4 trials being densely conducted in countries such as Germany, France, and the US, whereas earlier phases were located in Eastern Europe and the Baltics. Virtually no trials were conducted in countries with medium or low HDI.

The study has limitations. Lower MS incidence in Africa, Asia, and South America^5^ and lower Healthcare Access and Quality Index in these regions^6^ might have affected trial feasibility. Geographical and socioeconomic data were snapshots from 2019 to 2023 and thus might not reflect the development history of countries or regions. All trial data were obtained from ClinicalTrials.gov.

We found that studies used to demonstrate the efficacy of drugs for all human beings worldwide included only a small proportion of people living in countries with high HDI. Although this practice might be partly due to difficulties in conducting trials in countries with low HDI and lower MS incidence, the regional distribution of phase 4 trials suggests commercial interests of sponsors. Safety might be compromised by adverse effects that are unlikely in countries with high HDI but might occur in countries with low HDI, as they are virtually untested for. Excluding entire continents or socioeconomic strata from trials further exacerbates global inequities in access to treatment and introduces a bias for study interpretation on a global scale.

## Notes

Supplement 1.
eMethods.

eReferences

Supplement 2.
Data Sharing Statement
