# Supplementary material for: Socioeconomic and Regional Disparities in Industry-Sponsored Clinical Trials in Multiple Sclerosis
Source: JAMA Netw Open. 2023 Nov 29;6(11):e2345619. doi: 10.1001/jamanetworkopen.2023.45619 (PMC10687656; doi:10.1001/jamanetworkopen.2023.45619)
Supplement: Supplement 1. — eMethods. eReferences [file jamanetwopen-e2345619-s001.pdf]

Supplemental Online Content

Marti S, Hoepner AGF, Hammer H, Salmen A, Chan A, Hoepner R. Socioeconomic and regional disparities in industry-sponsored clinical trials in multiple sclerosis. *JAMA Netw Open*. 2023;6(11):e2345619. doi:10.1001/jamanetworkopen.2023.45619

eMethods.

eReferences

This supplemental material has been provided by the authors to give readers additional information about their work.

© 2023 Marti S et al. *JAMA Network Open*

eMethods.

All analyses and visualizations are documented in the corresponding Jupyter notebooks on the project's GitHub page, at https://github.com/drstrupf/clinical-trial-landscape. The document at hand summarizes the most important steps of the analysis.

Data sources

Data on clinical trials was downloaded from clinicaltrials.gov[1] for the search term "Multiple Sclerosis" on May 17, 2023, with no other filters applied. For geographical and socioeconomic data, including population estimates, we downloaded the 1:110m Cultural Vectors dataset from naturalearthdata.com[2] on August 8, 2023. The Human Development Index (HDI) data was downloaded from UN data portal of the United Nations Statistics Division[3] on August 8, 2023. An interactive overview of HDI data is provided by the United Nations Development Programme.[4] All source data are provided in the data/source directory of the project's GitHub page.[5]

Data preprocessing

Trial data In a first step, we filtered the raw MS trial data from clinicaltrials.gov by a manually curated condition list in order to remove false positives from the first coarse search. The filter list is provided in the data/manual/clinicaltrials folder on the project's GitHub page. The intervention information was simplified to categories only (e.g. "DRUG", "BEHAVIORAL", "OTHER"). Trials with multiple phases were included once per phase. We extracted the year from the start date, primary completion date, and completion date. Then we extracted the countries from the site location addresses and flagged trials where the location information was not provided in detail ("many locations" only). For those trials with detailed location listing we computed the number of sites per country, where each location was considered a site. The code can be found in notebooks/ 01_preprocessing.ipynb on the project's GitHub page. In a second step, we filtered the data on funder type "Industry", intervention type "Drug", and study type "Interventional", and we dropped trials without detailed location listing and the single early phase 1 trial. Trials with sites in Hong Kong were assigned to China. The code is provided in notebooks/02_data_analysis.ipynb.

Naturalearth data We added the population estimate, economy level, and income group for Singapore manually (source: Wikipedia[6]), since it was missing in naturalearth data. Then we dropped the regions "Antarctica" and "Seven Seas" from the analysis, since there are no trial data for these

regions, and since their low population estimates affect the normalization per capita per economy level and income group only very slightly ($O(10^{-6})$ and $O(10^{-5})$, respectively). We combined the OECD and non-OECD high income groups into one single high income group, the G7 and non-G7 developed regions into one single developed region category, and the BRIC, MIKT, and G20 emerging regions into one single emerging region category. The corresponding code is provided on the project's GitHub page in notebooks/02_data_analysis.ipynb.

Human Development Index data We only used the most recent entry per country, and added data manually for countries missing in the data set. The manually curated HDI data can be found on the project's GitHub page in data/manual/socioeconomic/manual_hdi.csv, where the data source is provided in an additional column for each entry. We categorized countries by HDI level according to the classification provided by the UN[4].

Data analysis

The number of trials, trial sites, and population estimates per region or socioeconomic category were computed as the sum over all countries within a given group. We computed the expected number of trials for each region or category by multiplying the region or category's population estimate by the total number of trials or sites divided by the total population, i.e. we computed the number of trials or sites for a given group under the assumption that trials or sites are distributed equally among the global population. The corresponding code is provided on the project's GitHub page in tools/counters.py and notebooks/02_data_analysis.ipynb. The $\log_{10}$ disproportionality shown in Figure 1 of the main manuscript is $\log_{10}$(number of actual sites/number of expected sites).

Regression analyses

The linear regression shown in Figure 2C was done using estimation by ordinary least squares (OLS) provided by the statsmodels[7] package for Python. The 95% confidence intervals are based on Student's t distribution. We refer to the package's documentation for implementation details.

Data visualization

We used the geopandas[8], matplotlib[9], and seaborn[10] packages for the creation of heat maps and world maps. Plot functions are provided in tools/visualization.py, and their usage is demonstrated in

the Jupyter notebooks 03_heatmaps.ipynb, 04_worldmaps.ipynb, 05_regression.ipynb, and 06_figures_for_publication.ipynb.

© 2023 Marti S et al. *JAMA Network Open*

eReferences

1. U.S. Department of Health & Human Services, National Library of Medicine. ClinicalTrials.gov. Version v2.1.1. https://beta.clinicaltrials.gov/ (data downloaded May 17, 2023).

2. Natural Earth. 2023. https://www.naturalearthdata.com/ (data downloaded Aug 8, 2023).

3. United Nations Statistics Division. 2023. https://unstats.un.org/UNSDWebsite/ (data downloaded Aug 8, 2023).

4. United Nations Development Programme. 2023. https://hdr.undp.org/data-center/human-development-index#/indicies/HDI

5. https://github.com/drstrupf/clinical-trial-landscape

6. https://en.wikipedia.org/wiki/Singapore

7. https://www.statsmodels.org/devel/index.html

8. https://geopandas.org/en/stable/

9. https://matplotlib.org/

10. https://seaborn.pydata.org/

© 2023 Marti S et al. *JAMA Network Open*
