# Supplementary material for: Socioeconomic and Regional Disparities in Industry-Sponsored Clinical Trials in Multiple Sclerosis
Source: JAMA Netw Open. 2023 Nov 29;6(11):e2345619. doi: 10.1001/jamanetworkopen.2023.45619 (PMC10687656; doi:10.1001/jamanetworkopen.2023.45619)
Supplement: Supplement 2. — Data Sharing Statement [file jamanetwopen-e2345619-s002.pdf]

Data Sharing Statement

Marti. Socioeconomic and Regional Disparities in Industry-Sponsored Clinical Trials in Multiple Sclerosis. *JAMA Netw Open*. Published November 29, 2023.
doi:10.1001/jamanetworkopen.2023.45619

Data
Data available: Yes
Data types: Data (not involving human participants)
How to access data: https://github.com/drstrupf/clinical-trial-landscape
When available: beginning date: 08-29-2023

Supporting Documents
Document types: Statistical/analytic code
How to access documents: https://github.com/drstrupf/clinical-trial-landscape
When available: beginning date: 08-29-2023

Additional Information
Who can access the data: Data is publical available via https://github.com/drstrupf/clinical-trial-landscape
Types of analyses: Replication only
Mechanisms of data availability: Publicly available at https://github.com/drstrupf/clinical-trial-landscape (contact form for technical requests provided).
